# Changes in Novel Biomarkers for Protein Oxidation in Pork Patties under Different Cooking Methods

**DOI:** 10.3390/foods13071034

**Published:** 2024-03-28

**Authors:** Chuanyu Guo, Shouyin Wang, Xiaolei Jia, Jinfeng Pan, Xiuping Dong, Shengjie Li

**Affiliations:** 1 School of Food Science and Technology, Dalian Polytechnic University, Dalian 116034, China; guo13252965138@163.com (C.G.); 18838940818@163.com (S.W.); jiaxl1122@163.com (X.J.); panjf@dlpu.edu.cn (J.P.); dxiuping@163.com (X.D.); 2 SKL of Marine Food Processing & Safety Control, Dalian 116034, China; 3 National Engineering Research Center of Seafood, Dalian 116034, China

**Keywords:** protein carbonylation, total carbonyl, total thiol, α-aminoadipic semialdehyde, lysinonorleucine

## Abstract

The aim of this study was to assess the effectiveness of different biomarkers to identify the levels of protein oxidation in pork patties induced by assorted cooking methods. To achieve this purpose, pork patties prepared from *longissimus dorsi* were cooked using three methods (frying, steaming, and roasting) at different internal temperatures (60, 70, 80, and 90 °C). Traditional biomarkers including total carbonyl and total thiol and novel biomarkers including α-aminoadipic semialdehyde (AAS) and lysinonorleucine (LNL) were determined. Results demonstrated that total thiol and AAS were the most successful biomarkers in distinguishing the three cooking methods in relation to protein oxidation, with AAS being the most sensitive. Moreover, as indicated by the biomarkers of total thiol and AAS, frying caused the highest level of protein oxidation, while steaming resulted in the lowest level when pork patties were cooked to the internal temperatures of 70 or 80 °C.

## 1. Introduction

Meats are usually cooked using assorted methods before consumption, which impart beneficial effects in terms of palatability, flavor, nutritional value, and food safety [1,2]. However, thermal treatments can also promote oxidative reactions in meat, which could ultimately cause significant damage to meat constituents, such as proteins and lipids [3,4]. Though less noticeable than microbial deterioration or lipid oxidation, protein oxidation has been drawing increasing attention from meat scientists since it could influence protein functionality and meat quality [5,6]. Pork remains the most consumed type of meat in China and accounts for more than half of the total meat and poultry consumption [7]. Several traditional cooking methods have been commonly employed in China in meat processing and household meat preparation, such as frying, steaming, stewing, and braising [8]. Meanwhile, roasting is the most used cooking method in commercial processing and food service operations in Western countries [9], which is also becoming increasingly popular in China. The effects of various thermal treatments on meat quality and protein oxidation have long been researched but remain a hot topic due to their significant implications in the industry and consumers’ preferences. In particular, regarding the underlying mechanisms, heat treatment has been well documented to trigger protein oxidation via the production of free radicals and the destruction of endogenous antioxidants [3,4,10]. Upon cooking, the extent of oxidative damage to proteins is highly dependent on the different extents of the heating process to which meat is subjected [11]. In general, the level of protein oxidation may be determined by the temperature level and duration of the heat treatment, where elevated protein oxidation occurs at higher cooking temperatures and extended times; however, it remains unknown if the influence of different cooking methods on the level of protein oxidation in cooked meat is based solely on the level of temperature and the length of time for which meat is exposed to heat treatment or if the prevailing mechanisms of these cooking methods also play a role [10].

From a methodological perspective, to elucidate this question, it is of great importance to find sensitive and reliable biomarkers to distinguish the differences in protein oxidation of meat cooked using different methods. The 2,4-dinitrophenylhydrazine (DNPH) method for the determination of the total carbonyl and the 5,5′-dithiobis-2-nitrobenzoic acid (DTNB) method for the determination of the total thiol have been widely used as traditional biomarkers to evaluate the level of protein oxidation in meat and meat products. However, the DNPH method has also been shown to interfere with the presence of malondialdehyde (a secondary lipid oxidation product), which can cause an overestimation of protein oxidation [12]. Moreover, this method has also been criticized for its non-specificity since it is limited to revealing information about the chemical structure and the mechanism of the formation of protein carbonyls [13]. Under this context, α-aminoadipic semialdehyde (AAS), an oxidative deamination product of lysine residues, has been highlighted as a novel, reliable, and more specific biomarker for protein oxidation in recent decades [14,15]. Besides AAS, lysinonorleucine (LNL), a Schiff base-type protein cross-link could also be used as a potential and specific biomarker to indicate the level of protein oxidation, and its quantification method has also been established recently [15]. The analysis of these novel biomarkers would increase our understanding of the chemical mechanism and pathways of protein oxidation under different cooking methods.

The present study aimed to compare the sensitivity of various biomarkers for protein oxidation and to find out the suitable biomarkers that can detect the difference among various cooking methods (steaming, frying, and roasting) in relation to protein oxidation.

## 2. Materials and Methods

### 2.1. Chemicals and Materials

Acetonitrile and formic acid were of high-performance liquid chromatography (HPLC) grade obtained from Sigma-Aldrich (St. Louis, MI, USA). *p*-aminobenzoic acid (ABA), 4-morpholineethanesulfonic acid (MES), and trichloroacetic acid (TCA) were purchased from Aladdin (Shanghai, China). BCA kit for protein concentration determination was obtained from Solabao Technology company (Beijing, China). AAS-ABA was previously prepared in our lab, and LNL was purchased from Santa Cruz Biotechnology (Dallas, TX, USA). Other chemicals were of analytical grade.

One commercial crossbreed pig (male, carcass weight of 70 kg) was slaughtered at a local slaughterhouse, and both sides of the *longissimus dorsi* muscles were obtained within 48 h postmortem. Meat samples were transported to the laboratory on ice, and the connective tissues and visible fat were then removed. Afterward, the meat was cut into small pieces, vacuum-packed, and stored at −20 °C for use within 2 months.

### 2.2. Sample Preparation

**Figure 1:**
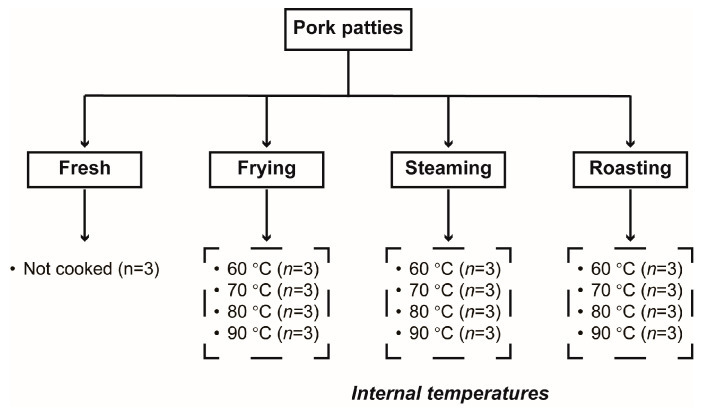
The experimental design.

The experiments of this study were repeated three times on three consecutive days, and for one batch of sample preparation, one proportion of the frozen meat was thawed at one time at 4 °C overnight, and pork patties were prepared and cooked on the same day. Thawed samples were ground with a meat grinder (TK-12, Shanghai Yingxiao Food Machinery Co., Ltd., Shanghai, China) using a 5 mm orifice plate, and the ground meat was molded into cubes of pork patties (3 × 2 × 2 cm) without the addition of salt, water, or any other ingredient. After preparation, pork patties were overwrapped with PVC film and stored in a refrigerator until cooking. The pork patties were then randomly assigned to different treatments, and the experimental design of this study was illustrated in Figure 1. For one batch of sample preparation, 39 pork patties were obtained, of which 3 were not cooked and were considered as the control group (Fresh). The remaining 36 pork patties were then randomly assigned to one of the following three cooking methods (12 pieces per cooking method) and cooked to the corresponding internal temperatures (three replicates per temperature point): (1) frying: pork patties were fried using a frying pan with 5 mL soybean oil at 230 °C, and both surfaces were heated during the cooking process; (2) steaming: pork patties were steamed using a combi oven (Model 101, RATIONAL AG, Landsberg am Lech, Freistaat Bayern, Germany) with water vapor at 100 °C in the “Steam” mode; and (3) roasting: pork patties were roasted using a combi oven (Model 101, RATIONAL AG, Otto Landsberg, Germany) set to 240 °C in the “Convection” mode. Before cooking, the internal temperatures of pork patties were allowed to equilibrate to room temperature of 22 °C. During the cooking process, the internal temperature was monitored using a digital thermometer (Testo thermocouple, Mod. 735-1, Lenzkirch, Germany) at the geometric center of the pork patties, and the heating process was considered complete when samples had reached the corresponding temperatures of 60, 70, 80, and 90 °C, respectively (70 or 80 °C were commonly required in meat processing and household meat preparation, and 60 and 90 °C were also chosen for comparison). Meanwhile, the duration of time elapsed from the beginning of cooking until the corresponding internal temperatures were obtained and also recorded for each cooking method. Results showed that the corresponding time lengths for frying to reach the internal temperatures of 60, 70, 80, and 90 °C were 160 s, 240 s, 300 s, and 390 s, respectively; for steaming, these time lengths were 210 s, 270 s, 360 s, and 600 s, respectively; for roasting, these time lengths were 360 s, 480 s, 600 s, and 720 s, respectively. After cooking, samples were cooled to room temperature, vacuum-packed, and stored at −80 °C until subsequent analysis.

For each cooking method, the heating rates and cook values were calculated. In particular, the degree of cooking at the center of pork patties was expressed in terms of cook value ($C_{T_{ref}}^{z}$) during the heating phase [16]. The cook value was calculated from the integration of the heat penetration curve,

(1) $$C_{T_{ref}}^{z}=\int_{0}^{t}10^{(T-T_{ref})/z}dt$$

where *t* is the time; *T_ref_* is the reference temperature and was set at 100 °C; *z* is the temperature increase that induces a 10-fold increase in the reaction rate of the chemical reaction taken as a reference and was set at 30 °C.

### 2.3. Determination of Total Carbonyl 

A 1 g quantity of pork patties was homogenized in 10 mL 0.15 M KCl at 1000 rpm for 30 s. Five aliquots (100 μL) of homogenate were mixed with 1 mL HCl–acetone solution (3:100, *v*/*v*) and centrifuged at 3000× *g* for 10 min. Afterward, 2 mL of 10% trichloroacetic acid (TCA) was added to the pellets, mixed, and centrifuged at 1200× *g* for 5 min (4 °C). Afterward, the total protein carbonyls were determined according to the DNPH method of a previous study [15]. Briefly, 0.4 mL of the protein pellets dissolved in 5% SDS were mixed with 0.8 mL of 0.3% (*w*/*v*) DNPH in 3 M HCl. After 30 min incubation at room temperature, proteins were precipitated, washed, and dissolved in 1.5 mL of 6 M guanidine hydrochloride in 20 mM sodium phosphate buffer (pH 6.5). The absorbance at 280 nm and 370 nm was measured with a UV-Vis spectrophotometer (UV 5200, Shanghai Metash Instruments Co., Ltd., Shanghai, China). The carbonyl content was then calculated and expressed as nmol/mg protein.

### 2.4. Total Thiol Determination

A 1 g quantity of pork patties was homogenized in 25 mL of 5% SDS in 0.1 M tris buffer (pH 8.0) at 1000 rpm for 30 s, and the total thiol content was determined according to the method of a previous study [17], with a few modifications. Briefly, 0.5 mL protein samples (1.5 mg/mL) were mixed with 2 mL 0.1 M Tris buffer (pH 8.0) and 0.5 mL DTNB reagent (10 mM DTNB in 20 mM Tris, pH 8.0) and incubated in the darkness for 30 min at room temperature. Afterward, the absorbance was measured at 412 nm using a UV-VIS Lambda 35 spectrophotometer (PerkinElmer, Waltham, MA, USA). The total thiol content was calculated and expressed as nmol/mg protein.

### 2.5. AAS and LNL Determination

Protein solutions obtained from Section 2.4 were diluted to 2 mg/mL, and the AAS and LNL contents were determined using the method described previously with small modifications [15]. In brief, precipitated proteins were dissolved in MES-SDS solution (4-morpholineethanesulfonic acid, 1% SDS, 1 mM DTPA, pH 6.0). Protein samples were then derivatized with 0.5 mL MES buffer containing 50 mM p-aminobenzoic acid (ABA) and 0.25 mL of 0.1 M NaCNBH_3_ in MES buffer at 37 °C for 90 min. After derivatization, proteins were precipitated with 10% TCA and washed with ethanol/diethyl ether (1:1, *v*/*v*). The protein pellets were then hydrolyzed in 6 N HCl at 110 °C for 18 h, and the hydrolysates were dried under constant nitrogen flow at 60 °C, followed by dissolving with 500 μL of Milli-Q water and filtered for HPLC-ESI-MS analysis. The HPLC-ESI-MS analysis was performed using a SHIMADZU Prominence LC-20A HPLC (SHIMADZU, Kyoto, Japan) coupled with a 4000QTRAP mass spectrometer (AB Sciex Pte. Ltd., Framingham, MA, USA). For the quantification of AAS and LNL, external standard calibration curves of both compounds were established.

### 2.6. Statistical Analysis

The experimental design was a completely randomized design with three replicates, and data were expressed as means ± standard deviation (SD). The statistical analysis was carried out using R (version 4.2.1, R Foundation for Statistical Computing, Vienna, Austria), and variance analysis was performed by two-way ANOVA with interaction. The least square means of different treatments were compared with TukeyHSD at the level of *p* < 0.05. Pearson correlation coefficients among cooking parameters and protein oxidation biomarkers were calculated using R to compare the sensitivity of different biomarkers for protein oxidation.

## 3. Results and Discussion

### 3.1. Differences in Heating Profile among Different Cooking Methods

**Figure 2:**
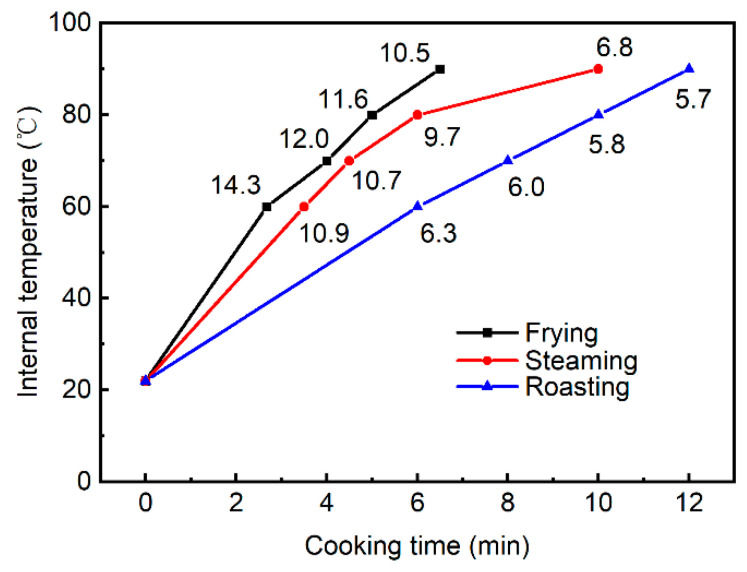
The profile of internal temperature and cooking time under different cooking methods. Numbers labeled indicate the heating rates at corresponding internal temperatures.

**Figure 3:**
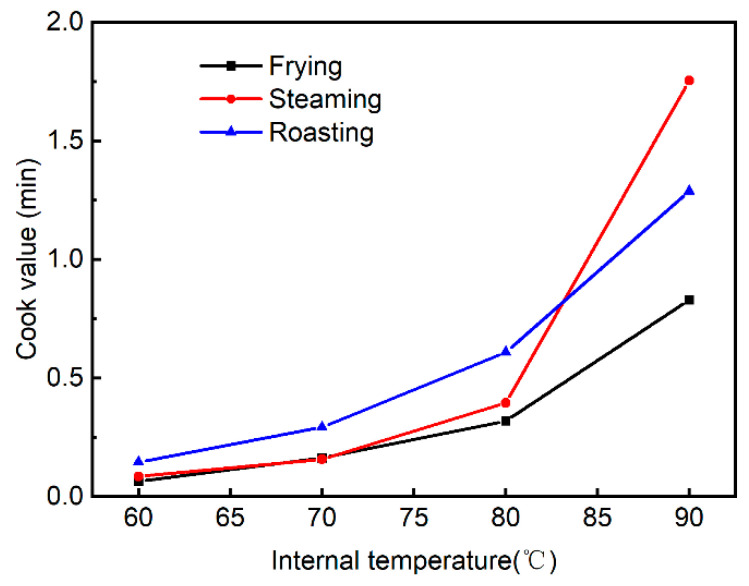
The cook values (equivalent min at 100 °C, z = 30) of different cooking methods at corresponding internal temperatures.

The three cooking methods applied in this study showed significant differences in heating profiles, which could be characterized by heating rates (Figure 2) and cook value (Figure 3). As shown in Figure 2, irrespective of the cooking method, the time required to reach corresponding internal temperatures is directly proportional to the internal temperature, and to obtain a higher internal temperature, a longer cooking time was required. Moreover, to reach the same internal temperatures, frying was observed to be the fastest, whereas roasting was the slowest. In particular, heating rates at the corresponding internal temperatures of different cooking methods were calculated, and it was shown that the heating rates were highest for the frying method (from 10.5 to 14.3 °C/min) and lowest for roasting (from 5.7 to 6.3 °C/min), and the heating rates for steaming were between 6.8 and 10.9 °C/min. The cooking methods of frying and roasting subject the food to indirect heat by surrounding the food with hot oil or air at higher temperatures (up to 300 °C), which differs from the steaming method using moist heat at lower temperatures (60 to 100 °C) [18]. In the present study, the pork patties were cooked at higher temperatures of 230/240 °C with the methods of frying and roasting, whereas pork patties were cooked at 100 °C with the method of steaming, and the changes in the heating rate of different cooking methods could be ascribed to their differences in the mechanisms of heat transfer and their different heat transfer coefficients. For frying, heat is transferred from the pan to the interior of the pork patties by conduction, while the combination of convection and conduction becomes responsible for the heat transfer for the methods of steaming and roasting. Moreover, the difference in heat transfer between steaming and roasting lies in that the media for convection are different (hot steam for steaming vs. hot air for roasting). Generally, these different mechanisms of heat transfer for different cooking methods lead to different heat transfer coefficients. The heat transfer coefficient of frying has been reported to be much higher than that of the oven cooking methods including both steaming and roasting [19,20]. Increased humidity in the oven cavity has been shown to cause increases in heat transfer coefficient, thus leading to a reduced cooking time, and the authors have explained that the steam present in the oven could release a large quantity of latent heat when condensing on the sample surface [21]. Therefore, it could be concluded that the heat transfer coefficient of steaming is higher than that of roasting but lower than that of frying, which led to the highest heating rate in the frying method and the lowest heating rate in the roasting method.

Cook values (CV) have been used to evaluate the heat intensities of various heat treatments with different time–temperature combinations, which can thus reflect the cumulative heat impact of time–temperature combinations on a food quality attribute [9,16,21,22]. In line with this, a higher cook value might indicate a higher level of oxidative damage to meat proteins, and thus, CV might correlate well with the changes in the contents of biomarkers for protein oxidation. Therefore, the cook values at the corresponding internal temperatures were further calculated to find out the difference in the heat intensities of different cooking methods (Figure 3). It should be noted that the CV values in the present study only represent the cumulative heat impact on the oxidative status of proteins located in the center of pork patties and not the cumulative heat impact on that of the whole piece of pork patty. Generally, contrary to the changes in heating rate, cook values were observed to be the highest in roasted samples and lowest in fried samples at the same internal temperature. In particular, when the internal temperature reached 60 or 70 °C, the roasting group had the highest CV, whereas the frying and steaming groups had lower CV. At 80 °C, CV was observed to be highest in the roasting group and lowest in the frying group. Consistently, it has been reported that the cook value of dry-air oven cooking (roasting) in the center of samples was higher than that of the steam cooking when the same internal temperature of 74 °C was obtained [9]. In addition, a significant increase in CV was found for the steaming group at 90 °C, making the CV of the steaming group the highest and the CV of the frying group still the lowest at 90 °C, and this might be explained by the smaller temperature difference between the cooking environment and the meat patties of the steaming group, leading to the drop in the heat transfer coefficient and the increase in the cooking time.

The quality characteristics of meat and meat products vary considerably depending on the types and intensities of the cooking methods applied [23]. For instance, different cooking methods could result in different proportions of heme and nonheme iron in meat [24,25], and both types of iron could promote protein oxidation to different extents [6]. In this sense, it is plausible to deduce that protein oxidation could be affected by the types and intensity of different cooking methods, and as a result of different mechanisms of heat transfer, different cooking methods could cause different extents of production of free radicals and destruction of endogenous antioxidants, and consequently, lead to different levels of protein oxidation. As mentioned above, the three cooking methods used in the present study could be featured by the differences in the heating rate and the cook value, where the method of frying was characterized by the highest heating rates and the lowest cook values at the same level of internal temperatures, and the method of roasting was observed to have the lowest heating rates but the highest cook values. Therefore, how these differences impact the changes in traditional and novel biomarkers for protein oxidation is further discussed in the following parts. By doing this, we intended to find out the most sensitive biomarkers that can detect the difference among various cooking methods in relation to protein oxidation.

### 3.2. The Traditional Biomarkers for Protein Oxidation

**Figure 4:**
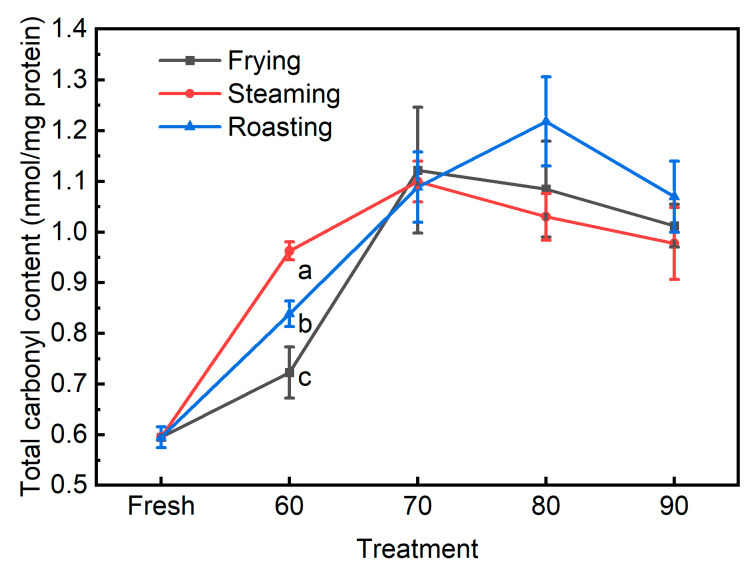
The total carbonyl contents in pork patties cooked to various internal temperatures using different cooking methods. Fresh: pork patties were not cooked; 60, 70, 80, and 90: pork patties were cooked to the internal temperature of 60, 70, 80, and 90 °C, respectively. Different letters for different cooking methods at the same temperature indicate significant differences at the level of *p* < 0.05.

It has been well accepted that heat treatment could trigger protein oxidation via the production of free radicals and the destruction of endogenous antioxidants, with more protein oxidation at higher cooking temperatures and extended times [3,4,10]. Herein, total carbonyl and total thiol were regarded as the common biomarkers for protein oxidation. The generation of carbonyls has been regarded as the most common damage for oxidized proteins during meat processing and storage, which is usually determined using the DNPH method [26]. Internal temperature and the interaction between the cooking method and the internal temperature showed significant effects on the total carbonyl content of cooked pork patties (Table 1 and Figure 4). The protein carbonyl content in the fresh patties was 0.6 nmol/mg protein, and irrespective of the cooking methods, the total carbonyl contents of the cooked pork patties were observed to be significantly higher than the fresh counterparts. Moreover, the total carbonyl contents increased with an internal temperature from 60 to 70 °C, and the values remained unchanged or even decreased when pork patties were cooked to higher internal temperatures. The present study also observed that total carbonyl content was positively correlated with internal temperature (*r* = 0.40, *p* < 0.05, Table 2), cooking time (*r* = 0.41, *p* < 0.05), and heating rate (−0.34, *p* < 0.05). However, the total carbonyl content was observed to be significantly different among the three cooking methods when pork patties were cooked to the internal temperature of 60 °C, with the lowest in frying and highest in steaming, and no such discrimination could be observed when pork patties were cooked to higher internal temperatures. At high internal temperatures, decreases in the total carbonyl content were noticed in all cooking methods, suggesting total carbonyl might not be a perfect indicator of protein oxidation at high internal temperatures. Though the DNPH method has been extensively used to determine the level of protein oxidation of assorted meat and meat products, it has also been pointed out that this method would overestimate the total amount of protein carbonyls due to the presence of accounting absorbance from artifacts, exceeding derivatization agent and lipid-derived carbonyls [12,27,28]. Accordingly, the present study showed that the roasted pork patties had lower values of TBARS than the steamed ones when cooked to the same internal temperatures (data not shown), which generally presented the opposite patterns of the oxidative status of proteins, indicating the occurrence of a chemical reaction between the secondary lipid oxidation products (for instance, MDA) and meat proteins. In line with this, Hu et al. [29] reported that relatively lower TBARS values were detected in roasted than steamed or boiled samples, which might be ascribed to the fact that the secondary lipid oxidation products can be involved in further reactions with other meat components, such as proteins. Therefore, total carbonyl might not be a suitable biomarker to discriminate the oxidative differences in proteins of pork patties heated using different cooking methods.

**Figure 5:**
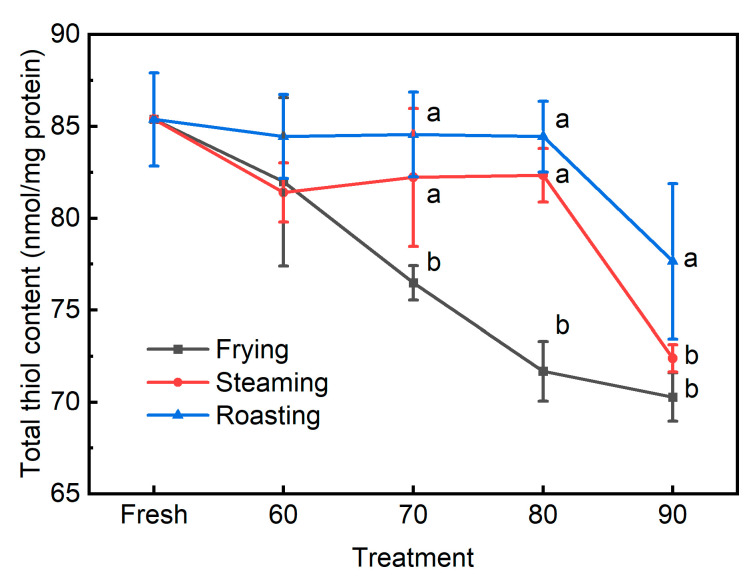
The total thiol contents in pork patties cooked to various internal temperatures using different cooking methods. Fresh: pork patties were not cooked; 60, 70, 80, and 90: pork patties were cooked to the internal temperature of 60, 70, 80, and 90 °C, respectively. Different letters for different cooking methods at the same temperature indicate significant differences at the level of *p* < 0.05.

Thiol-containing amino acid residues in the meat proteins (methionine and cysteine) are highly susceptible to reactive oxygen species (ROS) and can be oxidatively degraded via a chain reaction similar to lipid oxidation [30]. Therefore, the loss of the thiol groups has also been commonly regarded as an important traditional indicator for protein oxidation. Both the main effects and the interaction were found to significantly affect the total thiol contents of cooked pork patties (Table 1 and Figure 5). The total thiol content in the fresh patties was 85.4 nmol/mg protein for both the steaming and roasting groups, and the total thiol contents were not significantly decreased until the internal temperature reached 80 °C compared to the fresh patties, whereas for the frying groups, the total thiol content begun to decline since the internal temperature exceeded 60 °C. The thiol-containing amino acid residues (e.g., cysteine and methionine) are the most susceptible to assorted reactive oxygen species due to their sulfur atoms, whereas other amino acids need more stringent conditions to be oxidatively modified [26]. As shown earlier, the pork patties were heated at higher temperatures in frying than in steaming, and the frying method had a much higher heating rate than roasting. Therefore, these differences might lead to more severe damage to the thiol group in fried pork patties. Moreover, when meat patties were cooked to an internal temperature higher than 70 °C, cooking methods began to show significant effects on the thiol content. At 70 or 80 °C, a lower amount of total thiol was observed in the frying group than others, while at 90 °C, the roasting group had a higher thiol content than others, indicating the frying method could lead to more severe oxidative damage to the protein thiol groups compared to steaming and roasting when pork patties were cooked to similar internal temperatures. Therefore, together with the observation that the total thiol contents in the steamed and roasted pork patties were not significantly decreased until the internal temperature reached 90 °C, it is indicated that the environmental temperatures and heating rates greatly impact the loss of total thiol. Another possible explanation could be that the method of frying in this study used oil as a medium for heat transfer, and the oxidation products from lipids during the frying process can further reciprocally increase the oxidation of protein. For instance, lipid oxidation can produce free radicals, which have been observed to initiate protein oxidation, and the secondary products derived from lipid oxidation can also interact with the amino acid residues of proteins [26]. As shown in Table 2, total thiol was observed to be negatively correlated with internal temperature (*r* = −0.59, *p* < 0.001) and cook value (*r* = −0.48, *p* < 0.01), indicating that higher internal temperature and heat input could induce the oxidative loss of thiol groups. Overall, the total thiol could be regarded as a sensitive biomarker to differentiate the assorted cooking methods used in the present study.

### 3.3. The Novel Biomarkers for Protein Oxidation

**Figure 6:**
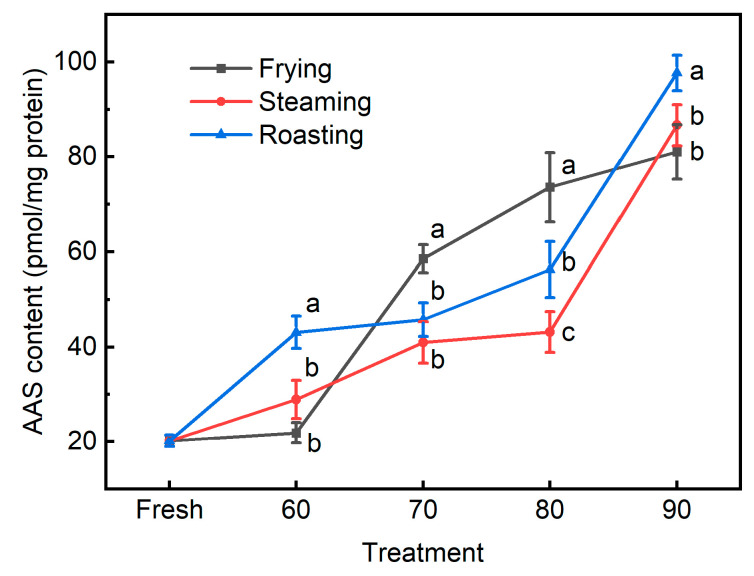
The AAS contents in pork patties cooked to various internal temperatures using different cooking methods. Fresh: pork patties were not cooked; 60, 70, 80, and 90: pork patties were cooked to the internal temperature of 60, 70, 80, and 90 °C, respectively. Different letters for different cooking methods at the same temperature indicate significant differences at the level of *p* < 0.05.

AAS has been regarded as a specific and reliable biomarker for protein carbonylation in muscle foods; furthermore, the determination of AAS could enable a better understanding of the pathways and mechanisms of protein carbonylation [14,27,31]. Depending on whether carbonyls are produced in proteins or introduced into proteins following their formation, primary carbonyls and secondary carbonyls can be clearly distinguished. AAS produced via the oxidative deamination of lysine residues of proteins belongs to the class of primary carbonyls, while secondary carbonyls are formed through the covalent bonding between protein and secondary lipid oxidation products [12]. In the present study, the formation of AAS was observed to be affected by internal temperatures and the interaction between the cooking method and internal temperature (Table 1 and Figure 6). The fresh patties contained 0.02 nmol/mg protein AAS, which was similar to those reported previously [15,32]. Irrespective of cooking methods, the AAS formation increased gradually with internal temperatures, and the highest values were observed in pork patties when the internal temperature reached 90 °C. Consistently, increases in AAS formation were also reported in pork treated with higher temperatures and longer times compared to raw meat [33]. When beef steaks were cooked to an internal temperature of 72 °C from 62 °C, the increased generation of AAS has also been noted in a previous study [3]. The accumulation of ROS and the continuous destruction of endogenous antioxidant systems induced by higher internal temperature or prolonged cooking time could also be the reasons for the increased formation of AAS with elevated internal temperatures in these three cooking methods. In general, the steamed pork patties were observed to contain lower levels of AAS than roasted pork when samples reached the same internal temperatures, which correlates well with the changes in the CV in these two groups. As shown in Figure 3, the roasting group had higher CV values than the steaming group at the internal temperatures of 60, 70, and 80 °C. Moreover, as illustrated by the correlation analysis (Table 2), AAS was shown to be significantly correlated with the cook value (*r* = 0.77, *p* < 0.001). As discussed earlier, a higher cook value might indicate a higher level of oxidative damage to meat proteins; therefore, the higher AAS levels in roasted pork patties than steamed counterparts could be attributed to the higher CV values, which could cause a more severe impact on the oxidation of lysine residues. Meanwhile, during the process of steaming, the water vapor would make direct contact with the meat surface and subsequently condense on it; therefore, a film of condensate could be formed on the surface of the meat samples [21], which might serve as a barrier against oxygen and thus prevent the oxidative damage to proteins, resulting in the lower extent of AAS formation in steamed samples than roasted ones. Another possible explanation might be that the roasting method applied a much higher environmental temperature than steaming, and the oxidation rate is higher at elevated temperatures [33]. Similarly, when sturgeon fillets were subjected to assorted cooking methods (boiling, steaming, microwaving, roasting, and deep-frying) until an internal temperature of 85 °C, roasted and fried ones presented a greater content and diversity of modifications of amino acids, including the oxidation of lysine to AAS [29]. However, this study further showed that fried pork patties generated the highest levels of AAS compared to the other cooking methods when cooked to the internal temperatures of 70 or 80 °C, though fried samples had lower CV values than the roasted ones. This observation indicates that frying could result in more oxidative damage to meat proteins than steaming and roasting when pork patties were cooked to 70 or 80 °C (commonly required in meat processing and household meat preparation), possibly because products of lipid peroxidation could further reciprocally increase the extent of protein oxidation. Moreover, AAS concentration was shown to be significantly correlated with other cooking parameters, such as internal temperature (*r* = 0.80, *p* < 0.001), cooking time (*r* = 0.68, *p* < 0.001), and heating rate (*r* = −0.45, *p* < 0.01), with stronger correlation coefficients than other indicators. Therefore, based on the aforementioned evidence, AAS might be regarded as the most sensitive and relevant indicator to distinguish the difference in the oxidative status of protein under assorted cooking methods.

**Figure 7:**
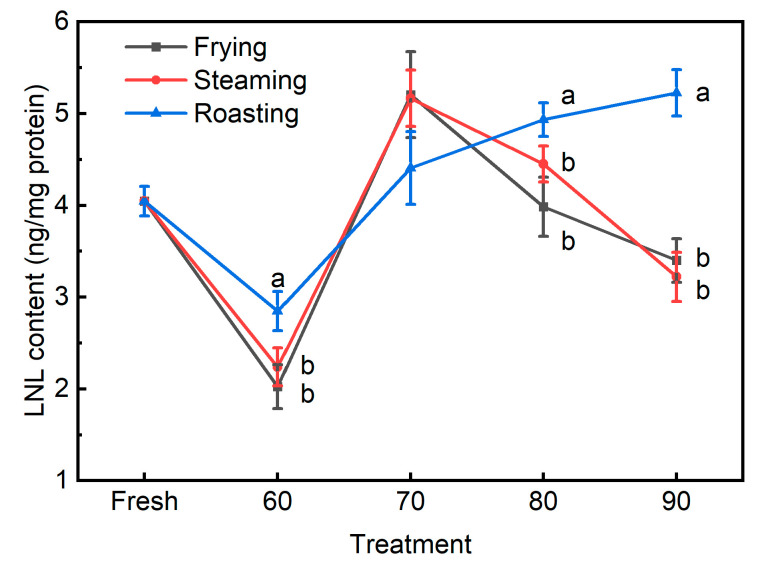
The LNL contents in pork patties cooked to various internal temperatures using different cooking methods. Fresh: pork patties were not cooked; 60, 70, 80, and 90: pork patties were cooked to the internal temperature of 60, 70, 80, and 90 °C, respectively. Different letters for different cooking methods at the same temperature indicate significant differences at the level of *p* < 0.05.

AAS formed in protein molecules can react with a lysine residue in a neighboring protein and form a covalent bond between proteins, which would be identified as lysinonorleucine (LNL) after acid hydrolysis [15,27]. Recently, an HPLC-MS/MS method for the quantification of LNL in meat and meat products has been developed and validated [15]. However, this previous study found that LNL was not a suitable protein carbonylation biomarker for different meat products, and it was explained that LNL is naturally present in collagen, and differences in their content could cause variations. Considering that the meat materials used in this study were identical for different cooking methods, LNL might be considered a potential biomarker for protein oxidation. In the present study, the LNL contents were significantly affected by the cooking method, internal temperature, and their interaction (Table 1 and Figure 7). Similar to the previous finding [15], it was confirmed in the present study that the LNL was naturally present in fresh pork patties. However, irrespective of the cooking methods, the LNL content declined when pork patties were cooked to the internal temperature of 60 °C. The reason might be that LNL is still chemically reactive and thus could be involved in further condensation reactions with other molecules [27]. Furthermore, when pork patties were roasted the LNL content increased gradually with internal temperature, whereas when pork patties were fried or steamed, it reached the highest level at an internal temperature of 70 °C. In particular, at the internal temperatures of 60, 80, and 90 °C, the roasted pork patties were observed to contain higher amounts of LNL than the other two groups. However, LNL was not observed to be correlated with any cooking parameters, such as internal temperature, cooking time, heating rate, and cook value. Therefore, it was indicated that the generation and degradation of LNL might occur concurrently during the heating process, and the level of LNL depends on which process prevails. In this sense, LNL might not be a suitable biomarker for protein oxidation in pork patties using assorted cooking methods. 

## 4. Conclusions

The present study demonstrated that total thiol and AAS were the suitable biomarkers to distinguish the three cooking methods in relation to protein oxidation, with AAS being the most sensitive, whereas total carbonyl and LNL were not indicated as being a proper biomarker to play such a role. Moreover, changes in total thiol and AAS suggested that, when pork patties were cooked to the internal temperatures of 70 or 80 °C, frying could cause more severe protein oxidation than steaming and roasting, with steaming resulting in the lowest level. The results from this study highlight the importance of selecting sensitive biomarkers to investigate the mechanisms underlying the effects of different cooking methods on protein oxidation. Considering the complexity of the chemistry behind protein oxidation, further research is warranted to develop more advanced methodologies to analyze the specific products of protein oxidation to understand better the impacts of different cooking methods on protein oxidation.

## Figures and Tables

**Table 1:** The statistical effects ^a^ of cooking method, internal temperature, and their interaction on the measured parameters.

	Cooking Method	Internal Temperature	Interaction
Total thiol	<0.001	<0.001	0.016
Total carbonyl	0.187	<0.001	0.028
AAS	0.113	<0.001	<0.006
LNL	<0.001	<0.001	<0.001
TBARS	<0.001	<0.001	<0.001

^a^ the *p*-values obtained from the two-way ANOVA in the statistical analysis.

**Table 2:** The Pearson correlation coefficients among cooking parameters and protein oxidation biomarkers.

	Cooking Time	Heating Rate	Cook Value	Total Thiol	Total Carbonyl	AAS	LNL
Internal temperature	0.71 ***	−0.36 *	0.83 ***	−0.59 ***	0.40 *	0.80 ***	0.22
Cooking time		−0.86 ***	0.81 ***	−0.08	0.41 *	0.68 ***	0.21
Heating rate			−0.52 **	−0.23	−0.34 *	−0.45 **	−0.19
Cook value				−0.48 **	0.27	0.77 ***	0.05
Total thiol					−0.11	−0.60 ***	0.04
Total carbonyl						0.40 *	0.51 **
AAS							0.07

*, <0.05; **, <0.01; ***, <0.001.

## Data Availability

The original contributions presented in the study are included in the article, further inquiries can be directed to the corresponding author.
